# Sane Persons in Asylums

**Published:** 1907-10-05

**Authors:** 


### SANE PERSONS IN ASYLUMS.

Periodically a newspaper scare arises about the confinement in asylums of persons who are quite sane, and there is an effervescence of sympathy, a declaration on the part of the public that "something should be done," and there, for the most part, the matter ends. Recently one of our contemporaries has come upon such a case, and the ex-patient himself gives his experiences, and makes a sensible suggestion. This is, that on the first onset of insanity the patient should be sent, not to one of the large asylums where chronic lunatics are kept, but to small convalescent homes by the sea, or in some other suitable place where, with care and good nursing, the attack might pass off, without his being subjected to the depressing influences of asylum life. It is quite likely that such homes would be very helpful in cases of temporary insanity arising from trouble and worry, or any of those causes which may comprehensibly shake the mental equilibrium for a time. If recovery took place the patient would have a better chance of regaining his old place in society if he had not to admit the fact that he had been at one time an inmate of an asylum—a statement which certainly creates a prejudice against the person who has to avow it. But while it may be true that cases are sometimes sent to an asylum which might be successfully treated elsewhere, it is not the case, as the unthinking public seem to imagine, that any large proportion of asylum inmates are sane or anything approaching to it. We can hardly call a person sane who behaves rationally only when protected from every anxiety of life. Yet every alienist will tell of cases which were to all appearance sane in the asylum, but broke down again as soon as they encountered the stress of daily existence. Such are at best mental invalids, and need to live in a sanatorium. Others are sane for long intervals, but are liable to sudden and severe outbursts of madness. It is certainly not desirable that the like of these should be free to go about the world. In fact, many tragedies are caused by such people, whose relations have too readily believed that they were cured when they were merely enjoying a lucid interval. One cannot but sympathise deeply with patients of this class, doomed through all the long dreary spaces when they are sane to consort with lunatics, because when the black fit is on them they are dangerous; but pity for their plight should not blind us to the risk involved in letting them go back into the world. That any number of sane people are permanently kept in an asylum is more than unlikely, for though, as the patient who gives his reminiscences remarks, it is of little use to depend on the annual visit of the Lunacy Commissioners—whose investigations must deal more with the conditions of the asylum than with the state of the patients—the medical superintendent and the other resident medical men are as anxious as could be desired to see their patients recover. The old legends of sane men imprisoned for life with cruel gaolers in order that unscrupulous relatives may enjoy their wealth survive now only in fiction of the cheaper kind. A patient may be kept for a few months after recovery, in order that the authorities may make as sure as they can that there is no danger of a relapse, and often a victim of periodic insanity must needs spend his good days as well as his bad ones in the asylum. Naturally he feels aggrieved, as do his friends if they visit him, finding him apparently in perfect health, and yet told that he is not fit to go home. If they had seen him as he had been a few days before, the idea that he had recovered would not have entered their minds, but he has no memory of the attack, and they did not witness it.

There is one serious warning in the ex-patient's statement. It deals with the discipline maintained by the attendants. Violent patients must of course be restrained, and in the course of this there is always the possibility of injury to the lunatic. Unfortunately the moanings of an insane patient may receive scant attention as being due rather to his mental than his physical condition; and it is also true that fellow-patients are afraid to speak of his sufferings from a nervous fear—characteristic of their weakness of mind—that they may anger the attendant. This throws on the medical men the greater responsibility, both of exercising a keen oversight on the attendants, and making a point of examining every patient who complains of pain. The pain may be imaginary in the majority of cases, but there is always the chance of its being real, and the knowledge that the complaint of even a lunatic will receive attention tends to make attendants cautious in using physical force. It has happened that an attendant has been charged with the manslaughter of a patient in an asylum, and even though the man was acquitted, such an incident could not but cast a stigma on the institution where it took place.

